# Combined treatment of isoflavone supplementation and exercise restores the changes in hepatic protein expression in ovariectomized rats - a proteomics approach

**DOI:** 10.1186/1550-2783-11-29

**Published:** 2014-06-14

**Authors:** Sun Yoon, Joomin Lee, Seung-Min Lee

**Affiliations:** 1 Department of Food and Nutrition, College of Human Ecology, Yonsei University, Seoul 120-749, South Korea; 2 Department of Food and Nutrition, College of Natural Sciences, Chosun University, Gwangju 501-759, South Korea

**Keywords:** Isoflavone, Exercise, Liver, Ovariectomy, Estrogen, Proteomics

## Abstract

**Background:**

Postmenopausal women experience adverse physiological changes caused by estrogen deprivation. Here, we hypothesized that the administration of isoflavone, a phytoestrogn, and/or physical exercise could reverse changes in the levels of hepatic enzymes disturbed by loss of estrogen to ameliorate postmenopause-related health problems.

**Methods:**

Thirty-week-old female Sprague–Dawley rats were divided into five groups: a sham-operated (SHAM) group, ovariectomized groups on a regular diet with exercise (EXE) and without exercise (OVX), and ovariectomized groups on an isoflavone supplemented diet with (ISO + EXE) and without exercise (ISO). Proteomic tools were employed to identify candidate hepatic proteins that were differentially expressed among the five animal groups.

**Results:**

INMT was detected in the SHAM but not in all of the ovariectomized rats. Seven proteins (PPIA, AKR1C3, ALDH2, PSME2, BUCS1, OTC, and GAMT) were identified to have differential expression among the groups. When compared to the SHAM group, the ovariectomy elevated the levels of PPIA, BUCS1, PSME2, AKR1C3, and GAMT while decreasing ALDH2 and OTC. Among these OVX-induced changes, OVX-increased BUCS1 and GAMT levels were noticeably decreased by ISO or EXE and further greatly down-regulated by ISO + EXE. In the case of PSME2, ISO and EXE further increased OVX-upregulated expression levels but ISO + EXE greatly reduced OVX-increased levels. On the other hand OVX-lowered OTC levels were elevated by ISO, EXE, or ISO + EXE. The protein levels of ALDH2, PPIA, and AKR1C3 were not significantly reverted by ISO, EXE or ISO + EXE.

**Conclusion:**

The combination of an isoflavone diet and exercise partly reversed ovariectomy-induced changes in hepatic protein expression levels. Our data suggest that the combinatory regimen of isoflavone supplementation and exercise may be beneficial to menopausal women through modulating hepatic protein expression profiles.

## Background

Postmenopausal women experience physiological changes related to estrogen deprivation. For example, decreased circulating estrogen levels have shown to be associated with menopausal metabolic syndrome with increasing adiposity [1]. In a rat model of metabolic syndrome, ovariectomy worsened its symptoms [2]. Low estrogen levels can also result in systemic inflammation in postmenopausal women [3]. Besides these physiological changes, postmenopausal women also show a reduction in lean body mass, which can partly be explained by insufficient estrogen levels [4]. Estrogen replacement therapy has been attempted to reverse the changes caused by menopause in an effort to decrease its cardiovascular and thrombotic risks [5] and to preserve bone mineral density [6]. It appears that estrogen plays several significant roles in women’s health.

Despite its many advantages, estrogen therapy is cautiously prescribed to postmenopausal women due to the possible increase in the risk of developing endometrial and breast cancers. In lieu of hormone therapy, treatment with isoflavones, natural plant substances, has been attempted to mimic the effects of estrogen in postmenopausal women [7]. Genistein, a type of isoflavone, was demonstrated to be efficacious in coping with estrogen deprivation [8-10]. While providing protective effects against the loss of estrogen, isoflavones also delivered some level of protection against hormone-responsive diseases such as breast and prostate cancers [11]. The frequent consumption of soy products, which are rich in isoflavones, has been shown to be related with a lower prevalence of breast cancer [12]. In addition to providing estrogen-like activity, the high intake of dietary isoflavone also reduced the risks of developing metabolic disorders including cardiovascular diseases, diabetes, and obesity compared to the high intake of animal products [13-16]. In particular, postmenopausal women with type 2 diabetes who received dietary isoflavone supplementation showed significantly reduced fasting insulin levels, indicating improvement in their insulin resistance [17].

Exercise is another lifestyle factor that can easily be modulated to improve lipid profiles. Postmenopausal women are advised to exercise in order to reduce abdominal adiposity, which increases after menopause [18], and to preserve muscle mass [19]. Exercise was also shown to be effective in reducing systemic and low-grade inflammation [20], which is a hallmark of chronic metabolic disorders. When provided with an exercise intervention, postmenopausal women were shown to have successfully lowered their levels of c-reactive peptide, which indicated diminished systemic inflammation [21]. Despite the many health benefits of exercise for postmenopausal women [18-20], exercise can also increase the production of free radicals that damage tissue [22].

Our group has previously reported that ovariectomized rats that underwent an exercise intervention had significantly elevated DNA damage in their lymphocytes compared to those that did not receive exercise [23]. Therefore, even if exercise interventions could lower blood LDL-cholesterol and the atherogenic index, the exercise may need to be monitored to minimize possible DNA damage in cases of estrogen deficiency [23]. The amount of free radicals generated by exercise may be lowered by isoflavone supplementation [23] because isoflavone possesses strong antioxidant properties and can scavenge reactive oxygen species [24]. Indeed, postmenopausal women who consumed isoflavones showed decreased levels of serum F2-isoprostanes, indicators of oxidative stress, suggesting a role of isoflavones as antioxidants [13,25]. Furthermore, isoflavone and exercise were shown to have a synergistic effect in ovariectomized mice, increasing their lean body mass apart from lowering body fat mass [19]. However mechanistic aspects of the isoflavone supplementation along with exercise in terms of the regulation of gene expression related to these beneficial effects have not been elucidated.

Considering that the liver plays a key role in metabolizing nutrients, hormones, and toxicants, protein expression patterns in the liver could reflect diverse changes in the systemic regulation of metabolism. To gain an insight into global changes in the gene expression upon isoflavone supplementation and/or exercise, we utilized a non-hypothesis driven proteomic approach. We hypothesized that an isoflavone-supplemented diet in combination with exercise could modulate the menopause-induced changes in hepatic protein abundance back towards its state prior to the onset of menopause. We compared the changes in all of the protein expression levels according to isoflavone supplementation and/or exercise regimen. The hepatic protein expression patterns among the following five different groups were compared: sham-operated (SHAM), ovariectomized only (OVX), ovariectomized and then isoflavone-supplemented (ISO), ovariectomized and then exercised (EXE), and ovariectomized, isoflavone-supplemented, and exercised (ISO + EXE).

## Methods

### Animals

Thirty-week-old female Sprague–Dawley (SD) rats were purchased from the Korea Food and Drug Administration, Laboratory Animal Resources Division (Seoul, Korea). The animals were individually housed in a room that was maintained at 22 ± 1°C with 55 ± 3% humidity under a controlled 12 h/12 h light–dark cycle. A total of forty rats fed on a chow diet were randomly divided into five groups and were allowed to adjust to the housing environment for one week. Then one group was sham-operated on (SHAM; n = 8) and the remaining four groups (OVX, ISO, EXE, and ISO-EXE; n = 8 each) were ovariectomized. After two weeks of recovery, SHAM, OVX and EXE groups were put on a basal AIN76A diet whereas ISO and ISO + EXE groups were put on an isoflavone diet, which is an AIN76A diet supplemented with 0.76 g of isoflavones per 100 g of diet. All animals were fed for 12 weeks *ad libitum*. As for treadmill exercise for 12 weeks, the EXE group and the ISO + EXE group exercised four times a week on a treadmill. Before starting their exercise regimen the animals in the EXE and ISO-EXE groups were accustomed to running on a motor-driven treadmill. During the first week, the rats ran at a speed of 10 m/min on a treadmill without an incline for 10 min on each day of exercise. The rats were subsequently trained to run at a speed of 16 ~ 17 m/min for 20 min during the second week and then again at this pace for 30 min from the third week until the end of their exercise regimen [23]. The Committee on Animal Experimentation and Ethics of Yonsei University approved the animal protocols used in the study.

At the end of the experiment, the animals were euthanized by cardiac puncture under ketamine anesthesia. The liver of the rats was removed and a portion of the tissue was transferred into a plain plastic tube which was snap-frozen in liquid nitrogen. The liver samples were kept at −80°C until use.

### Sample preparation

Frozen liver tissue samples were homogenized in extraction buffer consisting of 7 M urea, 2 M thiourea, 4.5% (w:v) 3-[(3-cholamidopropyl)dimethyl-ammonio]-1-propanesulfonate (CHAPS), 40 mM Tris, 100 mM dithioerythryol (DTE), 0.5% carrier ampholytes, and a protease inhibitor cocktail (Sigma Aldrich, St. Louis, USA). The homogenate was centrifuged at 45,000 rpm for 45 min to remove tissue and cellular debris. The supernatants were collected and stored at −70°C. Protein concentrations of the tissue lysates were measured using the Bradford method.

### Two dimensional electrophoresis (2-DE) and image analysis

The samples were diluted to 350 μl with rehydration solution [9 M urea, 4% CHAPS, 100 mM dithiothreitol (DTT), 0.5% (v/v) IPG buffer, and trace amount of bromophenol blue]. Isoelectric focusing (IEF) was performed to separate proteins according to their isoelectric points using IPG strips (non-linear pH 3–10, Amersham Biosciences, UK) and Multiphor II, an apparatus designed for IEF analysis (Amersham-Pharmacia, Amersham, UK). The IPG strips were initially incubated overnight in a rehydration solution. Samples were then loaded onto IPG strips and IEF was performed at 20°C with a current of 0.05 mA for a total of 85 kVh. The IPG strips were equilibrated to reduce the disulfide linkages through the addition of 10 ml of equilibrating solution containing isopropanol and 2.6% tributyl phosphine (Fluka) and then were gently rocked for 25 min. Second-dimension electrophoresis was performed using 9-16% gradient gels and the Iso-DALT apparatus (Hoefer Scientific Instruments, San Francisco, CA), and was then stopped when the tracking dye reached the anode end of the gels.

The 2-DE gels were visualized by silver staining and scanned using a GS800 photometer (Bio-Rad). The digitized 2-DE gel images were analyzed with PDQUEST (GenBio, Geneva, Switzerland) and compared by the matching method. Differentially expressed spots were selected based on a minimum two-fold difference between the groups.

### In-gel tryptic digestion

Candidate spots were excised from the stained gel, destained with 0.1 M ammonium bicarbonate in 50% acetonitrile (Sigma), and dried using a SpeedVac SC110 (SavantHolbook, HY). The excised and dried gel was rehydrated in a solution containing 1 M DTT and 0.1 M ammonium bicarbonate (pH 7.8) at 56°C for 30 min. The gels were subsequently incubated in a solution containing 1% iodoacetamide and 0.1 M ammonium bicarbonate (pH 7.8) for an additional 30 min in the dark. Next, the gels were washed with 0.1 M ammonium bicarbonate in 50% acetonitrile and dried. The gels were then rehydrated and incubated in trypsin solution (Promega, Madison, WI) overnight at 37°C. The trypsinized peptide solutions were sonicated for 30 min.

### Matrix-assisted laser desorption/ionisation-time of flight mass spectrometry (MALDI-TOF MS)

The peptides generated by in-gel digestion were subjected to a desalting and concentration step on a μZipTipC18 column (Millipore, Bedford, MA). The peptide mixtures were analyzed using MALDI-TOF MS (Applied Biosystems, CA). 0.5 ml of digested peptide was placed on a MALDI sample plate with 0.5 ml of matrix solution. Analysis was performed on a Perseptive Biosystem Voyager-DE STR (Perseptive Biosystems, MA). Internal mass calibration was performed using autolytic fragments derived from trypsin digestion. Proteins were identified by peptide mass fingerprinting (PMF) with the search engine program MASCOT and ProFound. All searches were performed using a mass window between 0 and 100 kDa. The criteria for positive identification of proteins were set as follows: (i) at least four matching peptide masses, (ii) 50 ppm or better mass accuracy, and (iii) the *M*r and p*I* of the identified proteins matched the estimated values obtained from image analysis.

## Results

### Proteomic comparison of hepatic protein expressions among the animal groups

**Figure 1:**
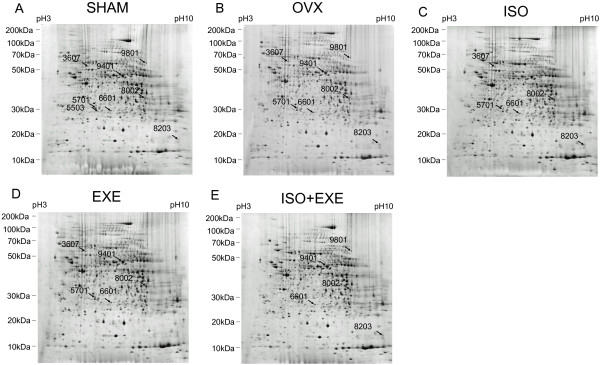
**Two-dimensional gel image analysis of the livers of ovariectomized rats following isoflavone supplementation and exercise.** Statistically significant spots are indicated by arrows in each gel. **(A)** SHAM group, sham-operated. **(B)** OVX group, ovariectomized. **(C)** ISO group, ovariectomized and provided isoflavone supplementation. **(D)** EXE group, ovariectomized and provided exercise training. **(E)** ISO + EXE group, ovariectomized and provided isoflavone supplementation and exercise training.

**Figure 2:**
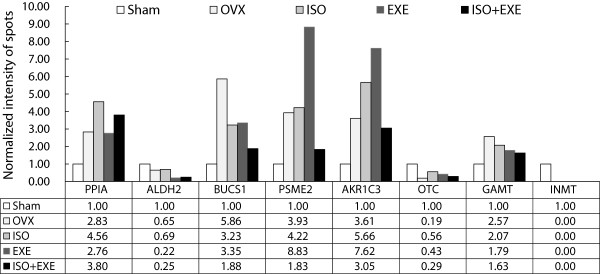
**Comparisons of protein spots differentially expressed in the livers of ovariectomized rats after isoflavone intake and exercise.** The normalized intensities of the protein spots in the five animal groups are shown.

Hepatic protein profiles in the animal groups are shown in Figure 1. After analyzing the gel images, differentially expressed spots were selected when their normalized spot intensities between the groups showed at least two-fold differences. The normalized protein spot intensities are presented in Figure 2. The proteins identified with differential expression levels are listed in Table 1. We identified eight differentially expressed proteins, which were spot number 5503 (Indolethylamine N-methyltransferase, INMT), 8203 (Cyclophilin A/peptidylprolyl isomerase A, PPIA), 3607 (butyryl coenzyme A synthetase 1, BUCS1), 5701 (proteasome activator rPA28 subunit beta, PSME2), 8002 (3 alpha-hydroxysteroid dehydrogenase, AKR1C3), 6601 (guanidinoacetate N-methyltransferase, GAMT), 9401 (aldehyde dehydrogenase, mitochondrial, ALDH2, and 9801 (ornithine carbamoyltransferase, OTC). The experimental ratios of molecular weights and isoelectric points (pI) matched those of theoretical data, suggesting that identification of proteins by our proteomic method was reliable. The sequence coverage is the percentage of the database protein sequence matched by the peptides identified in the proteomics. Our sequence coverage ranged from 9 to 71% for the identified proteins.

**Table 1:** Proteins differentially expressed in ovariectomized rat livers after isoflavone intake and exercise using MALDI-TOF MS/MS

**Spot number**	**Accession number**	**Official symbol**	**Protein identification**	**Theoretical MW(kDa)/pI**	**Measured MW(kDa)/pI**	**Score**^***a***^	**Coverage**
8203	NP_058797	PPIA	Peptidyl-prolyl cis-trans isomerase A	18.1/8.34	17.5/9.0	86	71
9401	P81178	ALDH2	Aldehyde dehydrogenase, mitochondrial	54.8/5.83	46.4/6.6	298	12
3607	NP_001101972	BUCS1	Butyryl Coenzyme A synthetase 1	27.8/5.57	50.1/5.1	39	13
5701	BAA08207	PSME2	Proteasome activator rPA28 subunit beta	27.1/5.52	30.1/5.3	75	13
8002	AAB19918	AKR1C3	3 alpha-hydroxysteroid dehydrogenase	37.6/7.03	36.1/7.4	121	9
9801	AAA41769	OTC	Ornithine carbamoyltransferase	39.9/9.1	52.1/7.8	220	14
5503	NP_001102492	INMT	Indolethylamine N-methyltransferase	30/5.7	29.3/5.4	99	12
6601	NP_036925	GAMT	Guanidinoacetate N-methyltransferase	26.7/5.69	28.2/5.8	48	16

^*a*^Score is −10xlog(*P*), where *P* is the probability that the observed match is a random event, based on the NCBInr database using the MASCOT searching program as MS/MS data.

### Comparison of hepatic protein expressions between sham-operated and ovariectomized rats

Hepatic protein profiles for each SHAM and OVX group are shown in Figure 1A and B. Spot number 5503 (INMT) was detected in the SHAM but not in any of the other ovariectomized groups (Figure 2). On the other hand, when compared to the SHAM, ovariectomized rats demonstrated an increase in protein levels, which were spot numbers 8203 (PPIA, 2.83 fold up), 3607 (BUCS1, 5.86 fold up), 5701 (PSME2, 3.93 fold up), 8002 (AKR1C3, 3.61 fold up), and 6601 (GAMT, 2.57 fold up). Two protein spots were down-regulated in ovariectomized rats when compared to the SHAM group, which include spot numbers 9401 (ALDH2, 1.54 fold down) and 9801 (OTC, 5.26 fold down).

### Effects of isoflavone supplementation on the levels of hepatic protein expression in ovariectomized rats

We also determined if isoflavone supplementation could affect protein expression patterns in ovariectomized rats. The expression of hepatic proteins in ovariectomized rats on an isoflavone-supplemented diet (ISO) was compared with that of the OVX group (Figure 1B and C). Isoflavone-supplementation resulted in the down-regulation of spot number 3607 (BUCS1, a 1.82 fold down) and the up-regulation of spot numbers 8203 (PPIA, 1.61 fold up), 8002 (AKR1C3, a 1.57 fold up), and 9801 (OTC, 2.95 fold up) compared to the rats without isoflavone supplementation (Figure 2). Spot numbers 9401 (ALDH2), 5701 (PSME2), 6601 (GAMT), and 5503 (INMT) did not change after isoflavone supplementation (Figure 2).

### Effects of exercise on the levels of hepatic protein expressions in ovariectomized rats

The influence of physical exercise was also examined in ovariectomized rats (Figure 1B and D). The animals that underwent regular exercise (EXE) demonstrated an up-regulation of spot numbers 5701 (PSME2, 2.25 fold up), 8002 (AKR1C3, 2.11 fold up), and 9801 (OTC, 2.26 fold up) in comparison with the OVX group. However, the EXE group showed a reduction in the protein expression levels of spot numbers 9401 (ALDH2, 2.95 fold down), 3607 (BUCS1, 1.75 fold down) and 6601 (GAMT, 1.44 fold down) compared to the OVX group. Exercise did not affect the expression of protein spot 8203 (PPIA) and spot 5503 (INMT) in comparison to the OVX group.

### Combined effects of both isoflavone supplementation and exercise on the levels of hepatic protein expressions in ovariectomized rats

Next, we examined if isoflavone supplementation and exercise had a combined effect on the hepatic protein expression profiles of ovariectomized rats (Figure 1B, C and E). The OVX-increased protein levels of spot number 3607 (BUCS1) was decreased markedly in the ISO + EXE group (3.12 fold down) whereas there were slight decreases in the ISO and the EXE groups (1.81 and 1.75 fold down, respectively) compared with that of the SHAM group. Similarly an elevation in the levels of spot 6601 (GAMT) in the OVX group (2.57 fold up compared to the SHAM) was decreased with a greater extent in the ISO + EXE group (0.63 fold down compared to the OVX) than those in either the ISO or the EXE. The ISO + EXE alone decreased the OVX-upregulated levels of spot number 5701 (PSME2) (2.15 fold down compared to the OVX). The OVX-increased protein levels of spot numbers 8002 (AKR1C3) were further elevated both in the ISO (1.57 fold up) and the EXE groups (2.11 fold up) but the ISO + EXE lowered the ISO or EXE-elevated levels of AKR1C3. The OVX-elevated expression levels of spot number 8203 (PPIA, 2.83 fold up compared to the SHAM) was slightly further increased in the ISO + EXE group (1.34 fold up compared to the OVX). On the other hand, spot number 9801 (OTC), which was down-regulated in the OVX, was elevated in the ISO + EXE group (1.53 fold up) but not as much as those in the ISO (2.95 fold up) and the EXE (2.26 fold up) compared to the OVX. The OVX-decreased levels of spot number 9401 (ALDH2) was not affected in the ISO but exhibited further reduction in the ISO + EXE group (2.95 fold down compared to the OVX), which was similar to the levels of the EXE.

## Discussion

Since the liver is the primary organ for processing nutrients, hormones, and drugs, we studied hepatic protein changes induced by ovariectomization in 30-week-old female rats employing proteomic tools. We also elucidated that ovariectomy-induced hepatic protein changes were effectively restored through a combination of isoflavone supplementation and exercise, which could benefit to combat the health conditions related to the loss of estrogen including the menopausal metabolic syndrome and osteoporosis. After ovariectomies, we identified that the proteins BUCS1, PSME2, AKR1C3, GAMT, OTC, ALDH2, PPIA, and INMT were differentially expressed in rat livers. These expression levels except INMT were further affected by isoflavone and/or exercise training.

BUCS1 is a mitochondrial enzyme that adds CoA to medium chain fatty acids for oxidative degradation and catalyzes the amino acid conjugation of many xenobiotic acids [26-28]. Elevated levels of BUCS1 in ovariectomized rats were dramatically reduced to the levels observed in the SHAM group by the combined regime of an isoflavone diet and exercise. The isoflavone diet and exercise demonstrated a slight reduction in the BUCS1 protein levels. It appears that the combinatory regimen of isoflavone diet and exercise seemed to be more effective than either intervention alone in blocking the abnormal activation of the oxidation of mitochondrial medium chain fatty acids resulting from the loss of estrogen.

PSME2, also known as proteasome activator (PA) 28 beta subunit, is part of the PA28αβ proteasome regulators found in immunoproteasomes [29] and has been implicated in the removal of proteins modified by oxidative stress [30]. A significant increase in the PSME2 protein levels in ovariectomized rats might indicate that the loss of estrogen increased the levels of proteins that were modified by oxidative stress. Indeed oxidative stress was reported to increase the expression levels of PA28αβ proteasome regulators [30]. Exercise alone further induced higher levels of PSME2 protein compared to what was observed in ovariectomized animals, which could indicate that exercise provided additional oxidative stress to the ovariectomized condition. While isoflavone intake did not change PSME2 protein levels, an abrupt reduction of PSME2 protein was observed when an isoflavone diet and exercise regimen was combined. It appears that isoflavone supplementation may be effective in decreasing oxidative stress in postmenopausal women who also regularly partake in physical exercise.

AKR1C3 (aldo-keto reductase family 1 member 3) is known to have steroid dehydrogenase activity (3α-HSD2, 17β-HSD5) [31], leading to the production of potent forms of testosterone and 17β-estradiol [31-33]. Furthermore, the over-expression of AKR1C3 was shown to be correlated with adiposity [34] and the size of the adipocyte [35]. The up-regulation of AKR1C3 in ovariectomized animals in comparison to the sham-operated animals might be a consequence of compensatory mechanism to increase the activity of sex hormones as a result of lack of estrogen and indicate an increased adiposity. Both an isoflavone diet and exercise further elevated AKR1C3 protein expression in ovariectomized rats. Interestingly the over-expression of AKR1C3 in the ISO and the EXE groups were reduced through a combined regimen of an isoflavone diet and exercise, thus avoiding the adverse effects of the hyper-activation of AKR1C3.

GAMT is an enzyme that catalyzes the methylation of guanidinoacetate acid, generating creatine and S-adenosylhomocysteine [36]. GAMT also plays a role in maintaining low levels of guanidinoacetate which is neurotoxic [37]. The overiectomy-induced increase in GAMT protein might be a protective mechanism to remove guanidinoacetate which could be increased by an estrogen-deficient condition. The reduction of ovariectomy-increased GAMT levels by exercise and further through the combination of isoflavone supplementation and exercise might indicate that the combined regime was more effective to lower the levels of quanidinoacetate followed by a reduction of GAMT than either exercise or isoflavone supplementation.

OTC is an ornithine carbamoyltransferase, a key enzyme in the urea cycle for removing ammonia, a byproduct of the breakdown of proteins in the body [38,39]. Compared to the SHAM group, the ovariectomized rats demonstrated a significant reduction in OTC protein abundance, which is consistent with the fact that OTC expression is regulated by estrogen at the transcriptional level [40]. In the present study, isoflavone supplementation or exercise alone significantly recovered OTC levels in ovariectomized rats to about 50% of that observed in the SHAM rats. This may suggest that either intervention is beneficial for maintaining the levels of OTC protein. Overall, the effects of an isoflavone diet and exercise on OTC protein expression seem to be beneficial.

PPIA acts as a molecular chaperone in protein folding and catalyzes the interconversion of peptidyl-prolyl imide bonds in peptide and protein substrates. The ovariectomy induced expression of PPIA was further increased by an isoflavone diet but was not affected by exercise, suggesting that the protective protein chaperone function might be induced by the loss of estrogen and further by isoflavone supplementation.

ALDH2 plays a crucial role in metabolizing acetaldehyde to acetic acid in the liver. ALDH2 protein reduces hepatotoxicity by decreasing the levels of acetaldehyde [41]. Deficiency in ALDH2 function caused the accumulation of lipid oxidants and osteoporosis [42]. ALDH2 protein levels reduced in the ovariectomized rats were further reduced in both the EXE and ISO + EXE groups. However, isoflavone supplementation alone had no effect on ALDH2 spot intensity. Thus, it appeared that exercise alone lowered ALDH2 protein expression in ovariectomized rats. Therefore, the loss of estrogen might increase acetaldehyde levels, resulting in an increased risk of oxidative stress and osteoporosis partly through the loss of ALDH2 protein levels. Exercise may reinforce the menopause-induced deficiency of ALDH2 protein levels.

INMT methylates tryptamine and structurally similar compounds [43]. Methylation is considered to be important in metabolizing endogenous and exogenous molecules such as drugs [43]. In the present study, the INMT protein spot was not detected in all of the ovariectomized groups. Neither isoflavone supplementation nor exercise was effective in recovering INMT protein expression. The absence of the INMT protein spot in the ovariectomized animals may indicate that estrogen is required for INMT expression and loss of estrogen hinder the methylation of tryptamine and related products due to insufficient INMT levels.

## Conclusions

Our proteomic data suggest that ovariectomy-induced changes in hepatic protein expression can be modulated by isoflavone supplementation or exercise. We have identified seven proteins differentially expressed depending on the treatment utilized: PPIA, AKR1C3, ALDH2, PSME2, BUCS1, OTC, and GAMT. The combination of an isoflavone diet and exercise was more effective in reversing the changes in ovariectomy-induced hepatic protein expression than either intervention alone. Thus, for women undergoing menopause, the combinatory regimen of isoflavone diet and exercise may be effective for adapting to a new estrogen-deficient condition and for protecting the body from stresses related to estrogen deprivation.

## Abbreviations

CTRL: Control; OVX: Ovariectomy; ISO: Isoflavone; EXE: Exercise; ISO-EXE: Isoflavone and exercise.

## Competing interests

Yoon S, Lee JM, and Lee SM report no competing interest.

## Authors’ contributions

YS contributed to the conception, design, analysis, and interpretation of the data. LJM made substantial contributions to the acquisition of the data. LSM contributed to the analysis and interpretation of the data as well as the critical revision and final approval of the manuscript. All authors read and approved the final manuscript.
